# Assessment of the Best FEA Failure Criteria (Part I): Investigation of the Biomechanical Behavior of PDL in Intact and Reduced Periodontium

**DOI:** 10.3390/ijerph191912424

**Published:** 2022-09-29

**Authors:** Radu Andrei Moga, Stefan Marius Buru, Cristian Doru Olteanu

**Affiliations:** 1 Department of Cariology, Endodontics and Oral Pathology, School of Dental Medicine, Iuliu Hatieganu University of Medicine and Pharmacy, Str. Motilor 33, 400001 Cluj-Napoca, Romania; 2 Department of Structural Mechanics, School of Civil Engineering, Technical University of Cluj-Napoca, Str. Memorandumului 28, 400114 Cluj-Napoca, Romania; 3 Department of Orthodontics, School of Dental Medicine, Iuliu Hatieganu University of Medicine and Pharmacy, Str. Avram Iancu 31, 400083 Cluj-Napoca, Romania

**Keywords:** periodontal breakdown, FEA failure criterion, maximum hydrostatic pressure, orthodontic movement, continuous orthodontic force, result accuracy

## Abstract

The accuracy of five failure criterions employed in the study of periodontal ligaments (PDL) during periodontal breakdown under orthodontic movements was assessed. Based on cone-beam computed tomography (CBCT) examinations, nine 3D models of the second lower premolar with intact periodontium were created and individually subjected to various levels of horizontal bone loss. 0.5 N of intrusion, extrusion, rotation, tipping, and translation was applied. A finite Elements Analysis (FEA) was performed, and stresses were quantitatively and qualitatively analyzed. In intact periodontium, Tresca and Von Mises (VM) stresses were lower than maximum physiological hydrostatic pressure (MHP), while maximum principal stress S1, minimum principal stress S3, and pressure were higher. In reduced periodontium, Tresca and VM stresses were lower than MHP for intrusion, extrusion, and the apical third of the periodontal ligament for the other movements. 0.5 N of rotation, translation and tipping induced cervical third stress exceeding MHP. Only Tresca (quantitatively more accurate) and VM are adequate for the study of PDL (resemblance to ductile), being qualitatively similar. A 0.5 N force seems safe in the intact periodontium, and for intrusion and extrusion up to 8 mm bone loss. The amount of force should be reduced to 0.1–0.2 N for rotation, 0.15–0.3 N for translation and 0.2–0.4 N for tipping in 4–8 mm periodontal breakdown. S1, S3, and pressure criteria provided only qualitative results.

## 1. Introduction

Finite Elements Analysis (FEA) became increasingly useful and accurate for investigating the periodontium when it is effectively used [1,2,3,4,5]. FEA is a mathematical-based method used for solving complicated problems in a multitude of fields including engineering and living tissue biology. This method subdivides a complex structure into much smaller and simpler parts called finite elements, assembled in a mesh of the object. Thus, this method allows a small and extremely complex living tissue like the periodontium and tooth to be divided into its components (i.e., dental pulp, neurovascular bundle [NVB], periodontal ligament [PDL], dentin, enamel, trabecular and cortical bone,) that can be independently analyzed. Thus, by evaluating the biomechanical changes, FEA supplies measurements of stress distribution inside the periodontium that are otherwise impossible to be performed in vivo [1,2,3,4,5]. FEA results must be validated directly experimentally (i.e., impossible for periodontal breakdown) and indirectly (i.e., corelating literature and in vivo results) [1,2,3,4]. A comparation between the amount of stress provided by the FEA analysis and maximum physiological hydrostatic pressure (MHP) of 2–16 KPa [2,3,4,6,7,8,9,10,11,12] represents a simple efficient validation criterion that should be mandatory [1,2,3,4,5,6]. Numerous FEA studies of PDL failed to employ an adequate failure criterion and reported results exceeding MHP that contradicted in vivo data [1,2,3,4,7,8,13,14,15,16,17,18]. 

When applying orthodontic treatment to periodontally compromised patients, the risks of further periodontal loss, NVB ischemia, pulp necrosis and orthodontic external root resorption could be avoided through a careful selection of the amount of applied force [2,3,4,5,6,7,8,10,11,12,17,18,19]. 0.25–3 N was reported to be safely applied in intact periodontium [5,6,10,11,12,19,20,21]. Recent indirectly validated FEA studies [2,3] reported (for intrusion, extrusion, rotation, translation and tipping of a lower premolar) a limit of 1.2 N for intact periodontium, 0.6 N for 4 mm loss, and 0.2–0.4 N for 8 mm reduced periodontium to be safely applied. Nonetheless, several other studies reported for upper and lower intact periodontium premolars 0.25–6 N of intrusive, tipping and translational forces to induce external orthodontic root resorption by exceeding the MHP and producing circulatory disturbances and periodontal loss [5,17,18]. Thus, there seems to be no consensus regarding the optimal orthodontic force magnitude safely used in the intact periodontium, while for the reduced periodontium the lack of evidence-based studies is important [2,3]. 

The biomechanical behavior of intact and reduced periodontium’s full assessment is possible only in vitro (i.e., based on CBCT-cone beam computed tomography) [5]. The qualitative results show stressed areas (i.e., prone to circulatory disturbances, ischemia, and further tissue loss), while the quantitative data allow the correlation with MHP and in vivo results [12]. The stress display also depends on the anatomy of structures (i.e., the need for accurate anatomical models) [12]. 

A FEA simulation has a major advantage over other methods by avoiding the random variability and supplying information about the stress distribution of each component instead of a single value for the entire structure (i.e., specific for in vivo and in vitro experiments [22]. FEA analysis allows accurate and predictable results if three conditions/limitations are acknowledged [12]. These are related to the employment of the adequate failure criterion (i.e., for ductile, or brittle type material [22]), to boundary conditions (i.e., linear-nonlinear analysis; isotropic-anisotropic and elastic-nonelastic properties), and the anatomical accuracy of the input data (i.e., high number of elements and nodes, fine grain mesh). Nonetheless, the accuracy of the results provided by a FEA simulation must be verified by performing a correlation with in vivo and in vitro existing data. There are numerous failure criteria to be used in the engineering field but in the living tissue biology (i.e., medicine and dentistry), the mostly used are Maximum Principal stress S1, Minimum Principal stress S3, Pressure/Hydrostatic Pressure, Von Mises (VM) and Tresca [2,3,4,5,6,7,8,9,10,11,12,13,14,15,16,17,18,19,20,21,22,23,24,25]. However, each of these five failure criteria has been designed to offer accurate results only for a specific type of material (i.e., ductile or brittle). Unfortunately, we found no evidence that previous studies [5,6,7,8,9,10,11,12,13,14,15,16,17,18,19,20,21,22,23,24,25] acknowledged this aspect in their methodology. A ductile material sustained deformation under stress before failure, while a brittle material fractures with little elastic deformation. Periodontal ligament, dental pulp and the neurovascular bundle should be considered closer to ductile materials [2,3,4,22]. Recent studies also employed hydrostatic Pressure failure criteria in the study of PDL [5,6,10,11,12,17,18]. However, this is a loading modality that results in little or no cellular deformation [23], contradicting existing knowledge of the biomechanical behavior of PDL.

Many FEA studies [12,13,14,15,16,24] of intact PDL employed the S1 (Max. Principal tensile stress) or S3 (Min. Principal compressive stress) failure criterions adequate only for brittle materials. These studies provided quantitative results higher than the 16 KPa reported for MHP and contradicted in vivo data [20,21]. Other studies [5,17,18] employed a hydrostatic pressure failure criterion and qualitatively correlated with in vivo data, nonetheless the reported results exceeded the MHP (e.g., TPa [17,18] and MPa [5] vs. KPa [2,3,4,6,7,8]). Some of these studies [6,10,11] suggested that the maximum safely limit for MHP should be 51.2 KPa. A few studies [12,13,14,25] employed a Von Mises (VM) failure criterion (i.e., adequate for ductile materials) in the intact periodontium, obtaining quantitative results lower than MHP, but without performing a correlation with the ischemic and external orthodontic cervical root resorption risks. Recent studies of our team introduced Tresca (adequate for ductile) failure criterion in the study of intact and reduced periodontium, reporting both Von Mises and Tresca criterions to provide both accurate quantitative and qualitative results for PDL, while S3 provided only qualitative data [2,3]. A recent study [12] limited to the intact periodontium suggested that compressive hydrostatic stress be the only criteria in the study of orthodontic root resorption and PDL, without any reference and discussion of the loading modality and failure criteria suitability. Thus, the need of a comparative study employing the mostly used failure criterions is obvious. To the best of our knowledge, there is no other FEA comparative study of all five types of failure criterions employed in the same FEA models, applying the same force and boundary conditions, and assessing the correlation between the force, stress, ischemia, further periodontal loss, and orthodontic external root resorption risks. 

In the current research flow, there is a limited number of reports describing a correlation between the induced force, the level of periodontal support, the orthodontic external root resorption risk and periodontal ischemia [2,3,4,6,10,11,12]. The analysis therefore provides evidence to clarify the problem of employing the adequate failure criterion in the study of PDL and the amount of orthodontic force safely used in reduced periodontium up to an 8 mm loss. By doing so, a predictive therapeutical prognosis and treatment improvement outcome can be achieved [5].

Thus, the aims of this study were: (a) to evaluate the biomechanical behavior of the intact and reduced PDL by employing the most used FEA failure criterions (Tresca, Von Mises, Pressure, S1 and S3) during five types of orthodontic movements (intrusion, extrusion, rotation, translation and tipping at 0.5 N), (b) to assess which FEA failure criterion provides the most accurate quantitative and qualitative results for the study of PDL (additionally, the PDL resemblance to ductile materials), (c) to assess individually for each orthodontic movement the amount of force safe to be used at 4 mm and 8 mm bone loss for avoiding the risks of orthodontic external root resorption and further periodontal breakdown.

## 2. Materials and Methods

The assessment of the adequate FEA failure criterion to be employed in the study of PDL and the amount of optimal orthodontic force safely used in reduced periodontium up to 8 mm loss is part of a larger research project assessing the behavior of teeth and the periodontium during orthodontic treatment at various levels of periodontal breakdown. The methodology has been previously applied and described in three recent publications [2,3,4] (i.e., secondary analyses with different aims stepwise conducted in various studies of the same models). Additionally, for increasing the accuracy of the results, the 3D models have been further refined. The research protocol and methodology has been approved by the Ethical Committee of the Iuliu Hatieganu University of Medicine and Pharmacy (158/2.04.2018). 

Nine patients (mean age of 29.81 ± 1.45 years, six females, informed oral consent) who met the main inclusion conditions: reduced noninflamed periodontium (i.e., treated chronic periodontitis/stage II/III grade B periodontitis enrolled in supportive periodontal therapy), complete mandibular arches with molars and premolars having various levels of bone loss, and applying for orthodontic treatment, were included. The mandibular area comprising the first molar and two premolars, with various levels of bone loss (mostly limited to the cervical third of PDL) was radiologically investigated (cone-beam computed tomography [CBCT], ProMax 3DS-Planmeca, Finland; voxel size 0.075 mm). 

**Figure 1:**
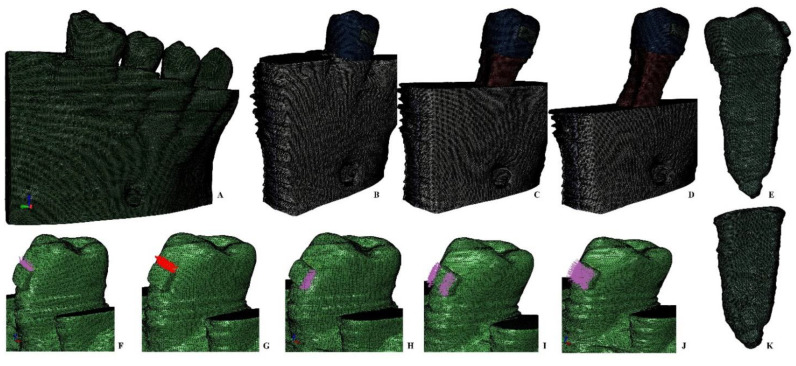
FEA mesh: (**A**)—one of the nine complete models (320 × 320 × 320 mm), (**B**)—second lower right premolar model (26 mm height) with intact periodontium, (**C**)—4 mm loss bone loss, (**D**)—8 mm bone loss, (**E**)—premolar (26 mm height), orthodontic loads vectors: (**F**)—intrusion, (**G**)—extrusion, (**H**)—translation, (**I**)—rotation, (**J**)—tipping, (**K**)—intact PDL (19.2 mm height).

**Figure 2:**
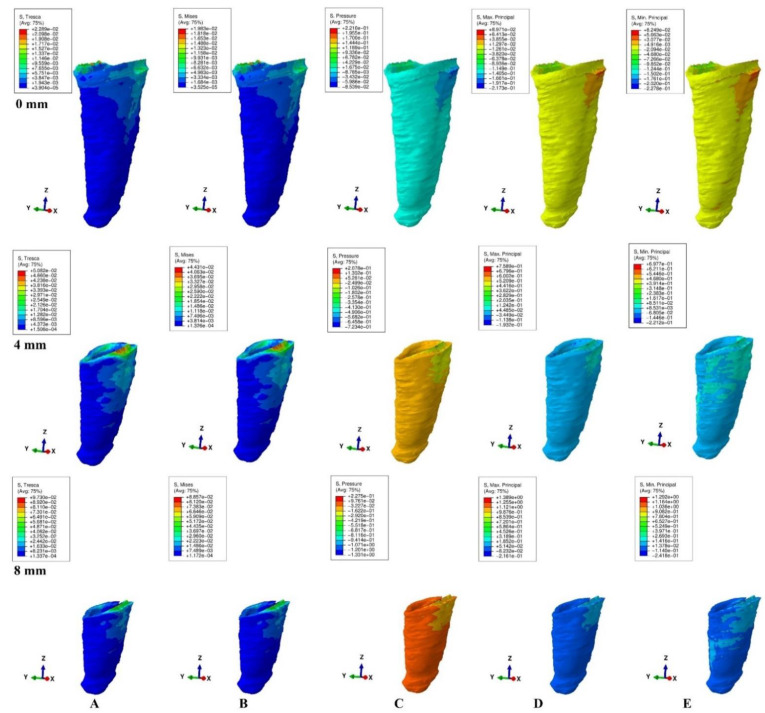
Stress distribution produced by rotation movement in the PDL of one of the nine models (intact, 4 mm and 8 mm reduced periodontium, vestibular-mesial view) in MPa: (**A**)—Tresca, (**B**)—Von Mises, (**C**)—Pressure, (**D**)—Max. principal, (**E**)—Min. principal.

**Figure 3:**
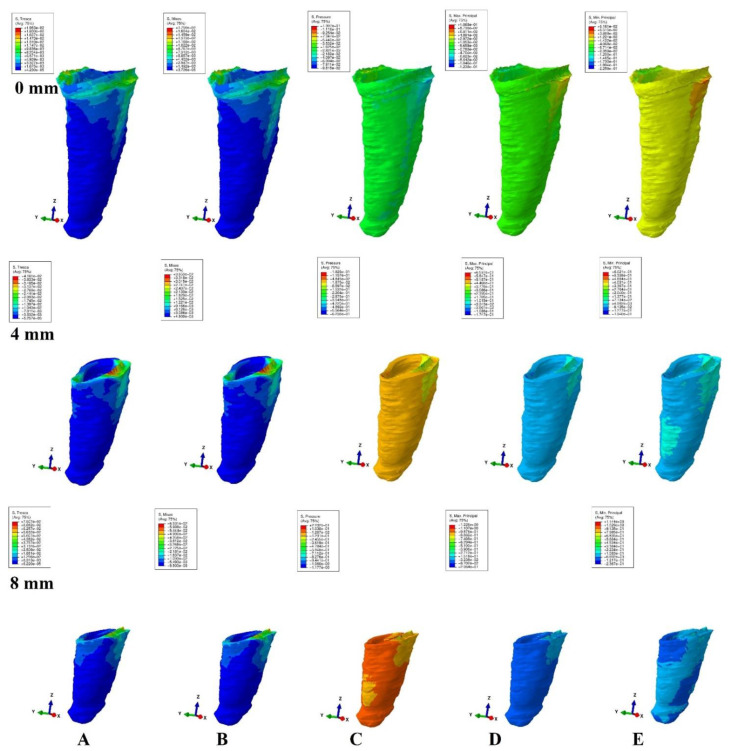
Stress distribution produced by translation movement in the PDL of one of the nine models (intact, 4 mm and 8 mm reduced periodontium, vestibular-mesial view) in MPa: (**A**)—Tresca, (**B**)—Von Mises, (**C**)—Pressure, (**D**)—Max. principal, (**E**)—Min. principal.

**Figure 4:**
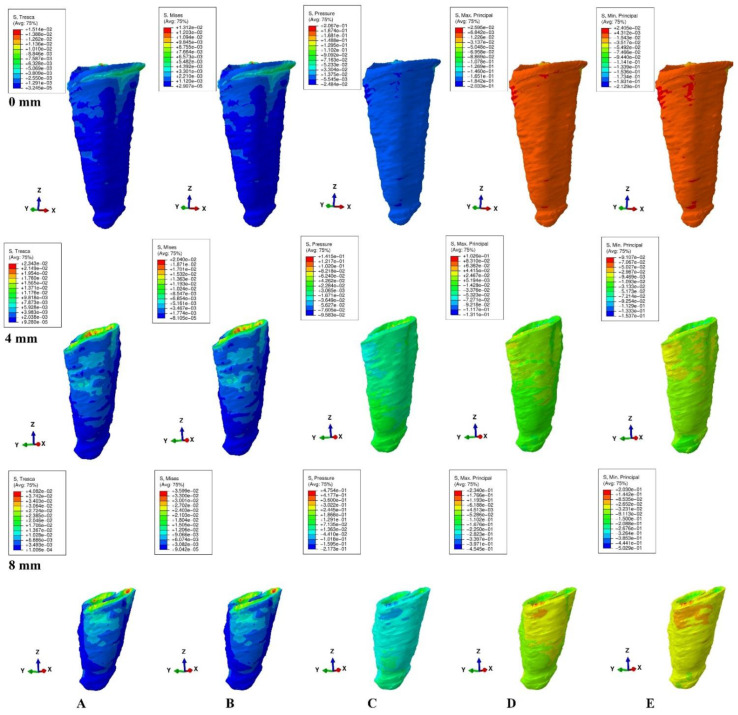
Stress distribution produced by tipping movement in the PDL of one of the nine models (intact, 4 mm and 8 mm reduced periodontium, vestibular-mesial view) in MPa: (**A**)—Tresca, (**B**)—Von Mises, (**C**)—Pressure, (**D**)—Max. principal, (**E**)—Min. principal.

**Figure 5:**
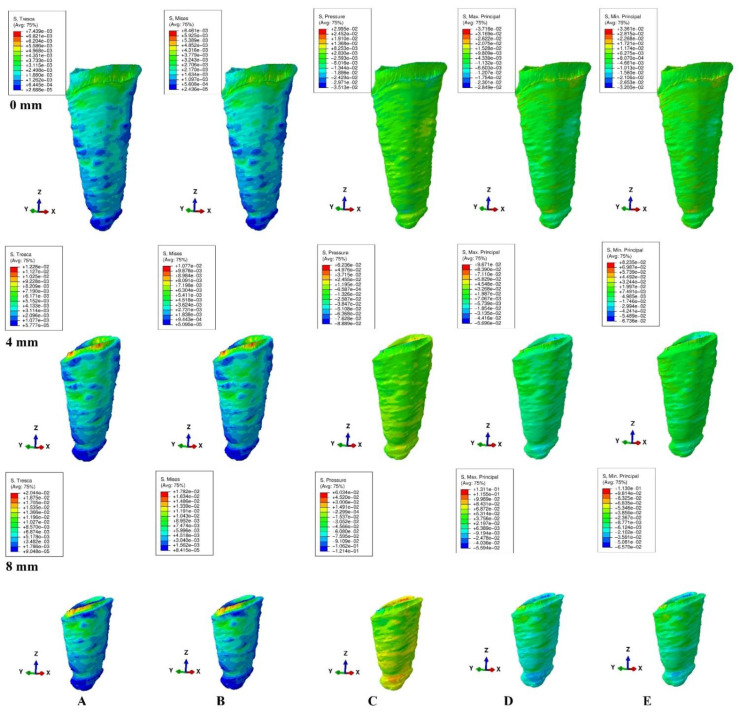
Stress distribution produced by extrusion movement in the PDL of one of the nine models (intact, 4 mm and 8 mm reduced periodontium, vestibular-mesial view) in MPa: (**A**)—Tresca, (**B**)—Von Mises, (**C**)—Pressure, (**D**)—Max. principal, (**E**)—Min. principal.

**Figure 6:**
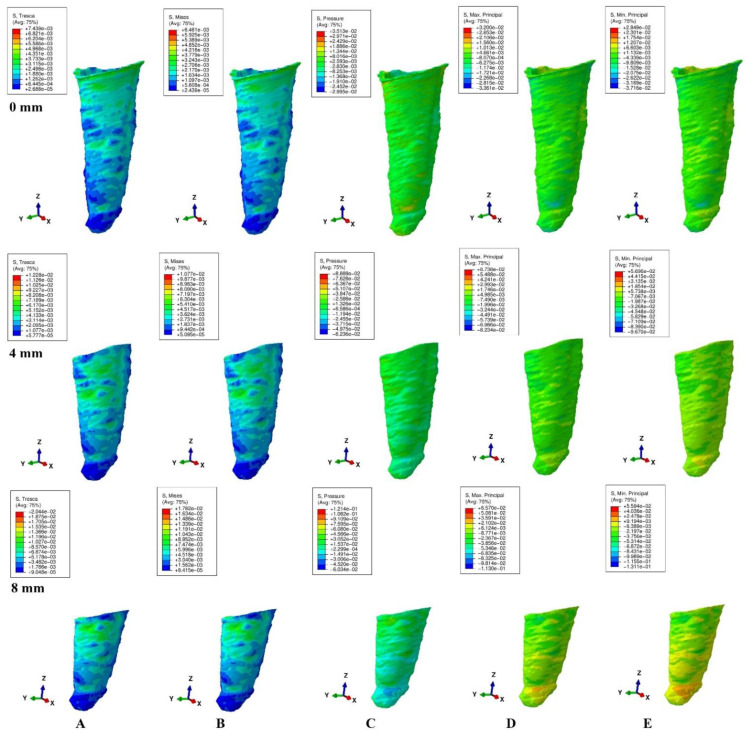
Stress distribution produced by intrusion movement in the PDL of one of the nine models (intact, 4 mm and 8 mm reduced periodontium, vestibular-mesial view) in MPa: (**A**)—Tresca, (**B**)—Von Mises, (**C**)—Pressure, (**D**)—Max. principal, (**E**)—Min. principal.

All reconstructions were based on the CBCT data and had been performed using AMIRA 5.4.0 software (AMIRA, version 5.4.0, Visage Imaging Inc. 300 Brickstone Square, Suite 201 Andover, MA 01810, USA). The 3D reconstructions of all the nine models included the second premolar (with different anatomical shapes and one or two roots). The components of the models (i.e., enamel, dentin, pulp, neuro-vascular bundle, trabecular and cortical bone, and bracket) had been manually reconstructed by the same person, employing the manual image segmentation technique for each slice (based on the different gray scale values and Hounsfield units). The manual segmentation technique allows for the reconstruction of areas that are not identified by the automated reconstruction algorithm (that refines most of the surfaces by simplification). Thus, the manual segmentation allowed a better anatomical accuracy and also an anatomical reconstruction of the missing tissues as close as possible to the anatomical original (i.e., patients displayed various levels of periodontal breakdown). The cementum was reconstructed as dentin due to similar mechanical properties. The PDL had a variable average thickness of 0.15–0.225 mm. After the manual reconstruction, nine 3D models with intact periodontium were developed containing the molar and two premolars (e.g., for being as precise as possible, Figure 1, Figure 2, Figure 3, Figure 4, Figure 5 and Figure 6 display one of the nine models, a two-rooted premolar with a particular hourglass shape). In each of the 3D models the molar and first premolar were then replaced with cortical and trabecular bone (i.e., using manual segmentation). Afterward, in each of the nine models, a gradual horizontal periodontal breakdown was simulated by manually reducing the bone and PDL by 1mm up to 8 mm of loss. Thus, from each of the nine models with intact periodontium, eight models with reduced periodontium were obtained (i.e., a total of 72 models), validated through meshing by AMIRA and ABAQUS software. Each software prevents the mesh creation and analysis if many surface elements anomalies are present, allowing only a limited number of anomalies that do not interfere with FEA analysis. The models with intact periodontium had 5.06–6.05 million C3D4 tetrahedral elements and 0.96–1.07 million nodes with a global element size of 0.08–0.116 mm (extremely fine grain mesh). Mesh convergence testing was performed for all models. Due to manual reconstruction, a limited number of surface anomalies and irregularities were present in all models (e.g., Figure 1 model, from a total of 6.05 million elements there were 264 element warnings), nonetheless the accuracy of the FEA was not altered (i.e., in areas with stress concentrations the models are quasi-continuous). Moreover, each software prevents the mesh creation and analysis if to many surface anomalies are present.

The models were subjected to FEA analysis in ABAQUS 6.11 software (Dassault Systèmes-France) by employing the mostly studied five failure criterions: VM, S1, S3, Pressure, and Tresca. Five of the most common pure orthodontic movements (intrusion, extrusion, translation, rotation, and tipping), under a continuous applied load of 0.5 N on the bracket surface, were simulated. Applying the force directly to the bracket and not to a modeled wire/arch (e.g., due to modelling difficulties, surface variability, angles, material properties) was considered adequate for simulating as best as possible the orthodontic effects and movements. However, it must be acknowledged that no simulation can recreate the complexity of in vivo orthodontic movement. Nonetheless, the complex study of the orthodontic effects can be achieved only by in vitro simulation, especially in the reduced periodontium. Homogeneity, isotropy, linear elasticity, and perfectly bonded interfaces were assumed as boundary conditions (Table 1). All components were assumed as free of boundary conditions while the base of the model had zero displacements. The structures were analyzed as a series of nodal displacements and the resulting stresses were calculated and displayed as color coded projections in the PDL of all models (e.g., Figure 2, Figure 3, Figure 4, Figure 5 and Figure 6, vestibular-mesial view). The cervical and apical third stress was quantitatively (i.e., average of the numerical values for each area of PDL) and qualitatively (i.e., color coded projections) assessed in all 72 models and displayed in Table 2 and Table 3, and Figure 2, Figure 3, Figure 4, Figure 5 and Figure 6. The quantitative average stress values for each of the five failure criterions for each of the movements were correlated with the reported maximum hydrostatic pressure (MHP) and the orthodontic external root resorption, and ischemic and further periodontal breakdown risks were assessed. Based on this risk assessment, the simulations with VM and Tresca failure criterions were redone for all models by reducing the amount of orthodontic force applied to the bracket to a level of 0.1–0.4 N, and then the average quantitative results were corelated once more with MHP. Stress increase speed was also assessed by performing a correlation analysis with average quantitative stress values for intact periodontium (in the apical and cervical third) as reference point for each orthodontic force and each level of periodontal breakdown.

## 3. Results

No significant differences between patients related to age, gender, periodontal status, or anatomy of the 3D models were found. In the assessment of the simulated periodontal breakdown (0–8 mm) for all five failure criterions applied to the orthodontic movements, the cervical third stress predominated (Table 3, Figure 2, Figure 3, Figure 4, Figure 5 and Figure 6). Quantitatively, in both intact and reduced periodontium, the predominant cervical third stress was higher than the apical and middle third stress (Table 2). Rotation showed the highest amount of stress followed in order by translation, tipping, extrusion, and intrusion (Table 2, Figure 2).

Quantitatively, in intact periodontium for 0.5 N of applied force, the Tresca and VM failure criterions displayed both apical and cervical amounts of stress lower than the 16 KPa reported for MHP (Table 2). The Tresca amount of stress was 1.15 (i.e., apically and cervically) times higher than the VM stress. 

The hydrostatic Pressure failure criterion displayed one of the highest amounts of stress among all five applied failure criterions along with S3, exceeding by far the reported MHP. The rotation, translation and tipping produced the highest amount of stress among the studied orthodontic movements. Thus, 0.5 N of rotation produced amounts of stress 4.12 times higher apically and 5.87 times higher cervically than the 16 KPa of MHP.

The S1 (tensile stress) and S3 (compressive stress) failure criterions displayed amounts of stress much higher than the Tresca and VM. All movements (except intrusion) produced in PDL amounts of stress exceeding the reported MHP. The rotation and translation movements displayed the highest amount of stress (S3, rotation, apically, 3.22 times higher; S1, rotation, cervically, 6.16 times higher) compared with the reported MHP. 

In reduced periodontium (up to 8 mm of bone loss) Tresca and VM displayed (both in apical and cervical third), and for rotation, translation, and tipping (only in apical third) amounts of stress lower than MHP for intrusion and extrusion. However, the cervical amount of stress produced by the last three movements exceeded 16KPa (MHP). In the cervical third of PDL 0.5 N of rotation, translation, and tipping produced a doubling of stress after 4 mm of bone loss, and a tripling after 5–6 mm. The Tresca amount of stress was 1.15 times higher than VM amounts of stress. Nonetheless, a simulation with 0.2 N of rotation, 0.3 N translation and 0.4 N of tipping by employing Treasca and VM produced in the cervical third of PDL an amount of stress under the MHP for a bone loss of up to 4 mm. The same simulation in a periodontal breakdown of 4 to 8 mm of loss produced a lower amount of stress than MHP for 0.1 N of rotation, 0.15 N of translation and 0.2 N of tipping. Up to a doubling of the stress, bone loss correlated directly proportionally with the force reduction for each movement type on one hand, and with the increase in shear and overall stress for cases with up to 8 mm periodontal breakdown on the other hand. Thus, a resemblance of PDL to ductile materials can be assumed.

The S3 and hydrostatic Pressure failure criterions displayed the highest amounts of stress among all five failure criterions for all orthodontic movements and exceeded by far the reported 16 KPa of MHP.

The S1 failure criterions displayed amounts of stress higher than MHP and the Tresca and VM stress values.

All five types of failure criteria displayed an expected correlation between the stress increase and the progress of periodontal breakdown for all five movements. 

Qualitatively, in intact periodontium, Tresca and VM criterions displayed almost identical stress distribution areas for all five movements (i.e., high cervical third stress for rotation, translation, and tipping; entire PDL for intrusion, and extrusion; (Figure 2)). The S1, S3 and Pressure criterions displayed a similar color-coded projection of the higher stress areas as VM and Tresca, with smaller extension and different color intensity (Table 3).

In reduced periodontium up to 8 mm of periodontal breakdown, Tresca and VM displayed the same stress distribution areas as in the intact periodontium (except for rotation and translation where areas of lower stress were also displayed in the apical third). The S1, S3, and Pressure failure criterions kept the same stress distribution for the intrusion, extrusion, and tipping movements. However, for rotation and translation, smaller areas of lower stress were displayed in the apical third of PDL.

## 4. Discussion

The herein comparative analysis is part of a larger research stepwise conducted [2,3,4] (study of periodontium under orthodontic forces) on continuously refined models assessing the adequate failure criterion appliable in the study of PDL for providing accurate results. Additionally, the amount of orthodontic force safely applied correlated with the level of periodontal breakdown and MHP was assessed. To the best of our knowledge, this is the only study of this type. 

PDL was largely studied (Table 4), under few orthodontic movements, using one (two [12,17,18]) anatomically simplified 3D models (i.e., upper 1st premolar [10,11,12,17,18], canine [6,10,11], 1st molar [24] and incisor [7,8,10,13,15,16]), with intact periodontium [6,10,11,12,17,18] or few levels of reduced periodontium (1, 2.5, 5, 6.5, 8 mm [15,16]; 2.5, 5, 6.5 mm [24]), employing failure criterions (pressure [5,6,10,11,12,17,18], S1 and/or S3 [7,8,14,15,16], and VM [12,13,14] failure criterions) without a correlation to the type of material for which they are suitable or MHP [7,8,13,14,15,16,25], employing both the non-linear [7,8,14] and linear [13,15,16,25] approach boundary conditions. Some of these studies reported quantitative results higher than MHP [3,5,7,8,14,15,16], forces safely applied up to 6 N [5,6,10,11,17,18] or the use of hydrostatic stress [12,17,18] as a single criteria for the study of PDL and orthodontic external root resorption.

The present analysis simulated a gradual horizontal breakdown (0–8 mm), in 81 3D models (i.e., up to 6.05 mil. elements and 1.06 mil nodes, PDL up to 1 mil. elements), and corelated the failure criterion with the suitable material type and the quantitative results with the MHP.

In intact periodontium, Tresca and VM failure criteria (adequate for ductile materials) displayed quantitative stresses lower that MHP for 0.5 N of applied force, in line with other reports [2,12,13,14,25]. The qualitative results are in agreement with some studies [2,13,25] but in disagreement with Roscoe et al. [12] unnatural display of stress. S1 and S3 (adequate for brittle materials) failure criteria produced quantitative values higher than MHP, in line with some reports [3,7,8,14,15,16], but in disagreement with others [7,8,12]. Pressure failure criteria for 0.5 N of applied force displayed stresses higher than MPH, in agreement with one report [5] but in disagreement with some others [5,6,10,11,12,17,18] (Table 4). 

In reduced periodontium (1–8 mm) Tresca and VM criteria displayed in the apical third of PDL quantitative stress values within the limits of MHP, in line with our previous study [2]. However, 0.5 N of the rotation, translation and tipping produced in the cervical third stresses exceeding the MHP. Nonetheless, by reducing the amount of applied force (i.e., 0.2–0.4 N for 4 mm loss and 0.1–0.2 N for 8 mm) the cervical third stress quantitative values remain within the MHP limits reducing the ischemia, further periodontal loss, and external root resorption risks. These results are in line with Proffit’s observations [20] and other reports [2,5,6,19,21], acknowledging the importance of prevention [2,3,4] vs. speed [12] of movement. S1 and S3 criteria displayed higher amounts of stress exceeding by far the MHP physiological limits, in line with other reports [3,15,16]. No correlation between the reduction of applied force and displayed stress could be established for these criteria. Pressure criteria displayed amounts of stress higher than MHP. However, no studies involving the reduced periodontium had been found for this criterion, thus a comparation was impossible.

As expected, the cervical third stress was found to be higher than the apical and middle third stress, in agreement with in vivo data. Among the five studied orthodontic movements, the rotation seems to be the most dangerous, showing the highest quantitative stress, closely followed by translation, in agreement with clinical data. However, it must be acknowledged that no other studies analyzing all five movements had been found, and thus a complete correlation between the results here and other studies was impossible to be determined.

The variations between the quantitative results herein and the results of previous studies (Table 4) may be due to the anatomical accuracy and structural complexity of the analyzed models, boundary conditions, amount and location of the applied force, and various levels of bone loss. Nevertheless, despite these differences, it seems that VM [13,14,25] and Pressure [5,17,18] quantitative reports are closer to the results found here than in the S1 and S3 reports [7,8,14,15,16].

Despite not being employed in the study of PDL, Tresca failure criteria is mathematically similar with VM. Compared with VM, Tresca provides results 15% higher, is more restrictive, and is prone to more accurate results due to lower stress limits.

FEA is accurate if limits are acknowledged. The complexity and variability of reduced periodontium means that its detailed study could be performed only by FEA analysis in vitro. Most of the PDL previous studies [5,17,18] involving the in vivo experimental part employed FEA analysis for the biomechanical behavior. The main FEA limitations are related to the employment of adequate failure criterion, boundary conditions simulating the anatomical behavior of the structures, and 3D models anatomically accurate. It also must be acknowledged that a FEA analysis could never reproduce the complexity of the clinical movements, and that in vivo there are no pure orthodontic movements. Nevertheless, FEA remains the only viable method when analyzing the periodontium.

The selection of the proper failure criterion is mandatory and is based on the type of material (i.e., brittle, or ductile) to be analyzed [22]. Human tissues cannot be simply divided into brittle or ductile, but rather have various resemblances to those characteristics [22]. To the best of our knowledge there are no studies (except ours [2,3,4]) to report such a classification of the periodontium components. The assumption that PDL has a close resemblance (by possessing some similar features) to ductile materials (but not being a traditional one) has been proven in this and a previous [2,3,4] analysis. Moreover, the successful employment of Tresca (i.e., non-smooth behavior by associating the ductile flow mode and the brittle fracture mode) as reliable failure criteria (proven by the qualitative and quantitative comparation with VM, Tresca being 15% higher) could provide more knowledge in the study of PDL. Tresca, being more restrictive than VM, is also more conservative because it predicts a narrower elastic region and agrees better with the experimental data, thus providing more accurate results. S1 and S3 failure criteria are acknowledged to work only for brittle materials [22]. From the biomechanical point of view, the use of Pressure failure criteria in PDL is debatable. The mechanism that causes yielding of ductile materials (PDL included) is shear deformation (VM and Tresca being consistent with this observation). Since there are no shear stresses for a state of hydrostatic stress, this component can be very large and still not contribute to yielding (only caused by the stresses which cause shape distortion) [23]. Moreover, Pressure criteria also seem to provide a similar pattern [12] with S3 in intact periodontium, in line with the results of this study.

The employment of adequate boundary conditions is important. The living tissues possess anisotropy, non-homogeneity, and non-linear elasticity [2,3,4,13,15,16,17,18,24]. Nonetheless, assuming all these in FEA is almost impossible, thus isotropy, homogeneity and linear elasticity are largely assumed when applying low forces under 1 N due to the simplicity of constitutive equations [12]. PDL was also regarded by some studies [6,10,11] as homogeneous hyper elastic-viscoelastic material and described using the Ogden hyper elastic model. However, under extremely low intensity loads (i.e., under 1 N), all materials are expected to exhibit linear elastic behavior [2,3,4], while the Ogden algorithm (designed for hyperelastic solids) is usually employed in handling large strains in rubber-like solids. Moreover, a 10–30% increase in stress for non-linear compared with the linear approach [2,3,4,7,8,14] had been reported, and thus the accuracy of results would not change. Nevertheless, in vivo the amounts of stress displayed by the periodontium could be lower than the results found here due to the association and combinations of different types of movements and anatomical differences. 

The anatomical accuracy of the analyzed model must be respected. Any alteration, idealization or simplification could significantly alter the studied parameters and alter the accuracy of the results and conclusions. Thus, the need to employ 3D models based on CBCT (large number of C3D4 or C3D10 elements and nodes) and not models created on idealized and simplified anatomy. Based on the methodology used here, the accuracy of the quantitative and qualitative results seems to be high. However, it must be acknowledged that the initial nine 3D models had various levels of bone loss. The lost bone and PDL had been manually reconstructed for obtaining nine models with intact periodontium, which could be considered as a shortcoming. However, this issue was expected, and we attempted to overcome it by using an experienced clinician when performing the reconstructions. 

The reduced periodontium had been insufficiently studied, and thus for a better understanding of its behavior under orthodontic forces, further studies are needed (e.g., dental pulp and neuro-vascular bundle biomechanical behavior in reduced periodontium under orthodontic forces as a continuation of the present study).

## 5. Conclusions

The present findings indicate that:

1. Only VM and Tresca criteria employment produced quantitative values lower than MHP up to 8 mm periodontal breakdown, which seemed to be adequate for the study of PDL (seeming to resemble more to ductile).
2. VM and Tresca criteria reported 0.5 N force, which appeared safe in the intact periodontium for all movements, and for intrusion and extrusion up to 8 mm bone loss. The amount of force should be reduced to 0.1–0.2 N for rotation, 0.15–0.3 N for translation and 0.2–0.4 N for tipping in 4–8 mm periodontal breakdown.
3. Tresca seems to be slightly more quantitatively accurate than VM (due to design specifications), while qualitatively they are similar.
4. S1, S3, and pressure criteria seems to only provide qualitative results for PDL.

## 6. Practitioner Points

When performing the orthodontic treatment in both intact and reduced periodontium, the ischemic risks and further complications (e.g., further bone loss and periodontal breakdown, dental pulp and neuro-vascular bundle ischemia and necrosis, internal and external orthodontic root resorption) must be acknowledged. Thus, for minimizing these risks, since optimal orthodontic force in the intact periodontium remains a subject of debate, and while little of the reduced periodontium is known, a specialist might consider the following observations:

1. In intact periodontium a continuous force of 0.5 N seems safe for all five orthodontic movements, while 8 mm reduced periodontium is safe only for extrusion and intrusion.
2. In a 4 mm reduced periodontium with 0.2 N of continuous rotation, 0.3 N of translation and 0.4 N of tipping are safe to be used, while at 8 mm of bone loss the applied force should be reduced to 0.1 N for rotation, 0.15 for translation and 0.2 N for tipping movements.

## Figures and Tables

**Table 1:** Elastic properties of materials table.

Material	Young’s Modulus, E (GPa)	Poisson Ratio, ʋ	Refs.
Enamel	80	0.33	[2,3,4]
Dentin/Cementum	18.6	0.31	[2,3,4]
Pulp	0.0021	0.45	[2,3,4]
PDL	0.0667	0.49	[2,3,4]
Cortical bone	14.5	0.323	[2,3,4]
Trabecular bone	1.37	0.3	[2,3,4]
Bracket (Cr-Co)	218	0.33	[2,3,4]

**Table 2:** Maximum stress average values (KPa) produced by orthodontic forces: a-apical; c-cervical.

Resorption (mm)			0	1	2	3	4	5	6	7	8
Intrusion	**Tresca**	a	**2.50**	**2.91**	**3.31**	**3.72**	**4.13**	**4.81**	**5.49**	**6.17**	**6.85**
0.5 N		c	**4.97**	**6.04**	**7.10**	**8.17**	**9.23**	**10.34**	**11.44**	**12.45**	**13.66**
	**VM**	a	**2.17**	**2.53**	**2.90**	**3.26**	**3.62**	**4.22**	**4.81**	**5.41**	**6.00**
		c	**4.32**	**5.26**	**6.21**	**7.15**	**8.09**	**9.05**	**10.00**	**10.95**	**11.91**
	Pressure	a	−13.68	−15.86	−18.40	−20.22	−24.55	−25.93	−27.31	−28.68	−30.06
		c	18.86	20.61	22.36	24.11	25.86	34.60	43.33	52.07	60.80
	S1	a	−6.28	−9.07	−11.85	−14.64	17.42	−18.32	−19.22	−20.12	21.02
		c	15.60	19.18	22.75	26.33	29.90	31.40	32.91	34.41	35.91
	S3	a	11.32	13.13	14.93	16.74	18.54	20.10	21.66	23.22	24.78
		c	12.07	13.69	15.31	16.92	18.54	20.10	21.66	23.22	24.78
Extrusion	**Tresca**	a	**2.50**	**2.91**	**3.31**	**3.72**	**4.13**	**4.81**	**5.49**	**6.17**	**6.87**
0.5 N		c	**5.59**	**7.01**	**8.43**	**9.85**	**11.27**	**13.01**	**15.50**	**17.10**	**18.75**
	**VM**	a	**2.17**	**2.53**	**2.90**	**3.26**	**3.62**	**4.22**	**4.81**	**5.41**	**6.00**
		c	**4.85**	**6.11**	**7.37**	**8.62**	**9.88**	**11.75**	**13.62**	**15.49**	**16.34**
	Pressure	a	13.68	15.86	18.40	20.22	24.55	25.93	27.31	28.68	30.00
		c	19.10	23.61	28.12	32.64	37.15	42.95	48.75	54.55	60.34
	S1	a	−6.60	−9.59	−12.57	−15.55	−18.54	−19.76	−20.98	−22.20	−24.78
		c	20.75	26.93	33.11	39.30	45.48	51.29	57.10	62.91	68.72
	S3	a	11.74	13.17	14.60	16.03	−17.46	−22.07	−26.69	−31.30	−35.91
		c	17.21	21.02	24.83	28.63	32.44	37.70	42.95	48.21	53.46
Translation	**Tresca**	a	**1.68**	**2.14**	**2.61**	**3.07**	**3.53**	**4.23**	**4.92**	**5.62**	**6.31**
0.5 N		c	**16.37**	**20.99**	**25.61**	**30.23**	**34.85**	**41.78**	**48.71**	**55.64**	**62.57**
	**VM**	a	**1.49**	**1.89**	**2.29**	**2.69**	**3.09**	**3.69**	**4.29**	**4.89**	**5.49**
		c	**14.59**	**18.55**	**22.52**	**26.49**	**30.45**	**36.44**	**42.45**	**48.44**	**54.44**
	Pressure	a	−28.21	33.27	38.33	43.39	48.45	62.29	76.27	90.11	103.80
		c	−79.11	−80.83	−82.54	−84.26	−85.97	−96.75	−107.53	−118.31	−129.10
	S1	a	10.53	17.06	23.60	30.13	−36.66	−49.25	−61.84	−74.43	−87.02
		c	48.91	62.01	75.21	88.35	101.50	114.08	126.65	139.23	151.80
	S3	a	38.69	41.50	44.31	47.11	49.92	64.52	79.11	93.71	108.30
		c	−93.59	104.62	115.65	126.67	137.70	159.13	180.55	201.98	223.40
Rotation	**Tresca**	a	**1.94**	**2.55**	**3.16**	**3.76**	**4.37**	**5.34**	**6.30**	**7.27**	**8.23**
0.5 N		c	**17.17**	**23.40**	**29.63**	**35.87**	**42.10**	**49.83**	**57.55**	**65.28**	**73.00**
	**VM**	a	**1.68**	**2.21**	**2.75**	**3.28**	**3.81**	**4.73**	**5.65**	**6.57**	**7.49**
		c	**14.80**	**20.34**	**25.88**	**31.41**	**36.95**	**44.33**	**51.71**	**50.83**	**66.46**
	Pressure	a	−34.32	38.89	43.47	48.04	52.61	63.86	75.11	86.36	97.61
		c	−85.30	−89.63	−93.95	−98.28	−102.60	−117.50	−132.40	−147.30	−162.20
	S1	a	12.90	18.30	23.70	29.09	−34.49	−46.45	−58.41	−70.36	−82.32
		c	64.10	79.13	94.16	109.18	124.20	139.40	154.60	169.80	185.00
	S3	a	56.60	59.46	62.33	65.19	−68.05	−86.29	−104.53	−122.76	−141.00
		c	−98.50	114.30	130.10	145.10	161.70	188.60	201.55	242.40	269.30
Tipping	**Tresca**	a	**1.29**	**1.96**	**2.64**	**3.31**	**3.98**	**4.71**	**5.44**	**6.16**	**6.89**
0.5 N		c	**11.36**	**13.89**	**16.43**	**18.96**	**21.49**	**25.44**	**29.43**	**33.41**	**37.42**
	**VM**	a	**1.12**	**1.71**	**2.30**	**2.88**	**3.47**	**4.12**	**4.77**	**5.42**	**6.07**
		c	**9.85**	**12.07**	**14.28**	**16.50**	**18.71**	**22.28**	**25.86**	**29.43**	**33.00**
	Pressure	a	13.75	16.03	18.31	20.59	22.84	34.97	47.09	59.22	71.34
		c	33.04	35.44	37.83	40.23	42.62	64.22	85.81	107.41	129.00
	S1	a	6.84	13.57	20.30	27.03	−33.76	−38.54	−43.31	−48.09	−52.86
		c	−31.37	39.43	47.50	55.56	63.62	77.54	91.46	105.38	119.30
	S3	a	24.05	30.97	37.89	44.81	−51.73	−61.62	−71.52	−81.41	−91.30
		c	−35.17	38.95	42.72	46.50	50.27	88.45	126.64	164.82	203.00

**Table 3:** Color-coded stress projection in PDL for different failure criteria.

Resorption (mm)		Intact Periodontium	8 mm Reduced Periodontium
Intrusion	Tresca	**A, M, C**	**A, M, C**
0.5 N	Von Mises	**A, M, C**	**A, M, C**
	Pressure	**A, M, C**	**A, M, C**
S1	Max. Princ.	**A, M, C**	**A, M, C**
S3	Min. Princ.	**A, M, C**	**A, M, C**
Extrusion	Tresca	**A, M, C**	**A, M, C**
0.5 N	Von Mises	**A, M, C**	**A, M, C**
	Pressure	**A, M, C**	**A, M, C**
S1	Max. Princ.	**A, M, C**	**A, M, C**
S3	Min. Princ.	**A, M, C**	**A, M, C**
Translation	Tresca	m, **C**	a, m, **C**
0.5 N	Von Mises	m, **C**	a, m, **C**
	Pressure	m, **C**	a, m, **C**
S1	Max. Princ.	m, **C**	a, m, **C**
S3	Min. Princ.	m, **C**	a, m, **C**
Rotation	Tresca	m, **C**	a, m, **C**
0.5 N	Von Mises	m, **C**	a, m, **C**
	Pressure	m, **C**	a, m, **C**
S1	Max. Princ.	m, **C**	a, m, **C**
S3	Min. Princ.	a, m, **C**	a, m, **C**
Tipping	Tresca	a, m, **C**	a, m, **C**
0.5 N	Von Mises	a, m, **C**	a, m, **C**
	Pressure	a, m, **C**	a, m, **C**
S1	Max. Princ.	a, m, **C**	a, m, **C**
S3	Min. Princ.	a, m, **C**	a, m, **C**

a-lower intensity apical third, m-lower intensity middle third, A-higher intensity apical third, M-higher intensity middle third, C-higher intensity cervical third.

**Table 4:** Stress display by different failure criteria in apical (a) and cervical (c) third of PDL.

Fail Criteria	Study	Force, Movement, Quantitative Stress, PDL Area
VM	Toms et al. (2003) [14], lower premolar, 5205 nodes, 1674 elem.	1 N extr., 8 KPa a, 7.75 KPa c
	intact periodontium	
	Merdji et al. (2013) [25], lower 1st molar, 557,974 elem.	10 N intr., 29.48 KPa a
	intact periodontium	3 N tip., 8.96 KPa a
		3 N transl., 6.78 KPa a
	Shaw et al. (2004) [13], upper incisor, 20,582 nodes, 11,924 elem.	extr. and intr., 2 KPa a
	intact periodontium	tipp., 1 KPa a
	Roscoe et al. (2021) [12], premolar, 1.67 mil. elem.	0.25 N intr. a and c 1.1 KPa
	intact periodontium	0.25 N tip., a and c 2.9 KPa
	Moga et al. (2022) [2], lower 2nd premolar,	0.2 N intr., 0.44 KPa a, 1.51 KPa c
	5.06–6.05 mil. elem. 0.96–1.07 mil. nodes	0.6 N extr., 1.33 KPa a, 5.18 KPa c
	intact periodontium	1.2 N transl., 3.58 KPa a, 28.06 KPa c
		0.6 N rot., 2.02 KPa a, 15.91 KPa c
		0.6 N tip., 1.34 KPa a, 10.52 KPa c
	reduced periodontium 8 mm	0.2 N intr., 1.22 KPa a, 4.76 KPa c
		0.6 N extr., 5.42 KPa a, 21.39 KPa c
		1.2 N transl., 26.28 KPa a, 117.00 KPa c
		0.6 N rot., 17.86 KPa a, 71.06 KPa c
		0.6 N tip., 7.29 KPa a, 43.19 KPa c
S1 and S3	Toms et al. (2003) [14], lower premolar, 5205 nodes, 1674 elem.	1 N extr., S1: 36.95 KPa a, −2.69 KPa c
	intact periodontium	1 N extr., S3: 28.49 KPa a, −11.6 KPa c
	Moga et al. (2021) [3], lower 2nd premolar	0.2 N intr., S3: −1.74 KPa a, −1.74 KPa c
	5.06–6.05 mil. elem. 0.96–1.07 mil. nodes	0.6 N extr., S3: 14.10 KPa a, 27.99 KPa c
	intact periodontium	1.2 N transl., S3: −97.79 KPa a, 93.03 KPa c
		0.6 N rot., S3: −56.27 KPa a, 68.07 KPa c
		0.6 N tip., S3: −18.53 KPa a, 28.89 KPa c
	reduced periodontium 8 mm	0.2 N intr., S3: −21.26 KPa a, −8.80 KPa c
		0.6 N extr., S3: 64.15 KPa a, 82.83 KPa c
		1.2 N transl., S3: −292.4 KPa a, 260.2 KPa c
		0.6 N rot., S3: −290.13 KPa a, 170.13 KPa c
		0.6 N tip., S3: −109.4 KPa a, −1023.49 KPa c
	Geramy et al. (2004) [15], upp. central incisor,	1.5 N tip., S1: 78.3 KPa a, −23.6 KPa c
	378,884 nodes, 32,768 elem., intact periodontium	1.5 N tip., S3:−74 KPa a, −28 KPa c
	reduced periodontium 8 mm	1.5 N tip., S1: 881KPa a, −395 KPa c
		1.5 N tip., S3: 740 KPa a, −491 KPa c
	Geramy et al. (2002) [16], upper central incisor,	1 N tip., S1: −37 KPa a, 55 KPa c
	726 nodes, 475 elem., intact periodontium	1 N tip., S3: −39 KPa a, −75 KPa c
		1 N intr., S1: 26 KPa a, −9 KPa c
		1 N intr., S3: −29 KPa a, −12 KPa c
	reduced periodontium 8 mm	1 N tip., S1: −440 KPa a, −288 KPa c
		1 N tip., S3: −475 KPa a, 300KPa c
		1 N intr., S1: –43 KPa a, 19 KPa c
		1 N intr., S3: –47 KPa a, −23 KPa c
	Hemanth et al. (2015) [7,8], upper central incisor,	0.2 N intr., S1: 1 KPa c
	239,666 nodes, 148,097 elem., intact periodontium	1 N tip., S1: −16.4 KPa a
		0.2 N intr., S3: −13.37 KPa a
		1 N tip., S3: 16.4 KPa a
	Roscoe et al. (2021) [12], premolar, 1.67 mil. elem.	0.25 N intr. a and c −5.3 KPa
	intact periodontium	0.25 N tip., a and c −7.3 KPa
Pressure	Hohmann et al. (2009) [17], 1st upper premolar	0.5 N intr., 4.7KPa−9.95 TPa a, 4.7 KPa c
	PDL 195,881 elem., tooth 711,114 elem., intact periodontium	
	Hohmann et al. (2007) [18], 1st upper premolar	3 N tip., 38.84KPa a, −68.09 KPa c
	PDL 152,776 elem., tooth 56,454 elem., intact periodontium	
	Wu et al. (2018) [6], upper canine	optimal force: tip. 0.28–0.44 N,transl. 1.1–1.37 N
	PDL 1263, elem., tooth 1928 elem., intact periodontium	rot. 1.7–2.1 N, extr. 0.38–0.4 N
	Wu et al. (2021) [10], lower incisor, canine, premolar	optimal force: rot. 2.2–2.3 N, 3–3.1 N, 2.8–2.9 N
	PDL 3032, 3416, 3851 elem., bone 5692 elem., intact periodontium	
	Wu et al. (2019) [11], upper canine	optimal force: intr. 0.8–0.9 N, extr. 2.3–2.6 N
	PDL 2272, elem., tooth 2101 elem., intact periodontium	
	Roscoe et al. (2021) [12], upper premolar, 1.67 mil. elem.	0.25 N intr. a and c −4.7 KPa
	intact periodontium	0.25 N tip., a and c −5.8 KPa
	Zhong et al. (2019) [5], lower 1st premolar, PDL 17575 elem.	0.25 N tip., a and c 10–20 KPa
	intact periodontium	
